# Which Theoretical Models or Frameworks Are Used to Understand Trauma‐Informed Care in Mental Health Settings? A Scoping Review

**DOI:** 10.1111/inm.70348

**Published:** 2026-09-11

**Authors:** Kim Jørgensen, Maria Estrup Scredi, Michael Haurum Marcussen

**Affiliations:** ^1^ Department of Health Services Research University of Southern Denmark Odense Denmark; ^2^ Department of People and Technology Roskilde University Roskilde Denmark; ^3^ Department of Cardiology Amager and Hvidovre Hospital Copenhagen Denmark

**Keywords:** implementation science, mental health services, organizational change, psychotraumatology, theoretical frameworks, trauma‐informed care

## Abstract

This scoping review examines and synthesizes the theoretical foundations underpinning trauma‐informed care (TIC) in mental health settings. A systematic search of CINAHL, Scopus, Web of Science, and PubMed yielded 1278 records, of which 23 peer‐reviewed studies published between 2010 and 2025 met the inclusion criteria. Reporting followed the PRISMA‐ScR (Preferred Reporting Items for Systematic reviews and Meta‐Analyses extension for Scoping Reviews) checklist and explanation. Seven overarching theoretical domains were identified: neurobiological and psychophysiological foundations, psychological and relational models, adverse childhood experiences and social determinants frameworks, SAMHSA and Harris & Fallot principles, socio‐ecological and intersectional frameworks, organizational and implementation science models, and integrated or hybrid approaches. These frameworks highlight the multidimensional nature of trauma and its implications for mental health nursing practice, interdisciplinary care, organizational change, and policy development. The findings underscore that TIC is not a single model but a pluralistic and evolving concept bridging individual, relational, and structural dimensions of trauma. Theoretical clarity can enhance implementation fidelity, reduce re‐traumatization, and support recovery‐oriented care. Future research should focus on evaluating hybrid frameworks and linking theoretical models to measurable individual, organizational, and system‐level outcomes.

## 1 Introduction

Trauma is increasingly recognized as a major influence on mental health and on experiences of distress across the life course. Exposure to traumatic experiences including interpersonal violence, childhood abuse, neglect, and systemic discrimination is strongly associated with elevated risks of depression, anxiety, post‐traumatic stress disorder (PTSD), substance use, and suicidality (Cloitre et al. 2011; Hughes et al. 2017). Beyond diagnostic categories, trauma shapes individuals' capacities for trust, safety, and engagement with mental health systems, with many service users describing feelings of disempowerment, stigma, or traumatization in encounters with mental health services (Sweeney et al. 2018). These challenges have contributed to calls for a paradigm shift in mental health services: moving beyond approaches that locate distress primarily within individual pathology toward approaches that recognize the pervasive impact of trauma and seek to foster recovery through safety, empowerment, and collaboration. Trauma‐informed care (TIC) has emerged in this context as a guiding framework for rethinking mental health practice and service delivery (Avery et al. 2021). TIC rests on the principle that trauma is not the exception but the expectation among people accessing mental health services (Sweeney et al. 2018). Instead of asking “What is wrong with you?”, TIC emphasizes “What happened to you?” (Bloom and Farragher 2013). In mental health settings, TIC is intended to prevent traumatization, enhance therapeutic alliances, and create organizational cultures that support both service users and staff. Core principles include physical and psychological safety, trustworthiness, peer involvement, collaboration, choice, empowerment, and recognition of cultural and historical trauma (Avery et al. 2021). These principles align with recovery‐oriented mental health policies that stress dignity, self‐determination, and person‐centeredness (Slade et al. 2014). The adoption of TIC in mental health care is evident across a range of contexts, including inpatient psychiatry, community mental health teams, and child and adolescent services (Bryson et al. 2017; Muskett 2014). Implementation efforts have included workforce training, adapting clinical environments, introducing peer support roles, and embedding trauma‐awareness in organizational policy. Evidence suggests that TIC can improve service users' experiences of care, increase staff satisfaction, and reduce the use of coercive practices such as seclusion and restraint (Isobel and Edwards 2017; Muskett 2014). Nevertheless, the integration of TIC into mental health systems remains uneven, and significant variation exists in how TIC is defined, operationalized, and evaluated (Thomas et al. 2019). One key source of variation stems from the theoretical foundations underpinning TIC. In mental health settings, multiple frameworks have been invoked to justify and guide TIC, often reflecting different disciplinary orientations. For instance, neurobiological theories such as the polyvagal theory explain the physiological underpinnings of trauma responses and their relevance for therapeutic relationships (Porges 2021). Psychological theories of attachment highlight the relational disruptions caused by early trauma and the importance of trust and security in recovery (Cloitre et al. 2011). Sociological perspectives, such as the adverse childhood experiences (ACEs) framework, emphasize the population‐level impact of trauma on mental health outcomes. Organizational and systems theories, such as relational coordination (Hoffer Gittell 2020) or implementation science models, focus on how mental health institutions can embed trauma‐awareness into service structures, policies, and team dynamics (Bryson et al. 2017). Despite this theoretical richness, there is currently no consolidated understanding of which models are most frequently used or most useful for conceptualizing TIC in mental health care. The study applies trauma‐informed care principles in a practical manner without explicitly grounding them in a theoretical framework (Muskett 2014). Others reference multiple frameworks simultaneously, leading to conceptual ambiguity. This lack of clarity complicates evaluation and comparison of TIC initiatives across different mental health services and may hinder the field's ability to demonstrate effectiveness and sustainability (Thomas et al. 2019). Furthermore, without explicit theoretical grounding, TIC risks being reduced to a set of aspirational principles rather than a coherent framework capable of informing meaningful change (Sweeney et al. 2018). Understanding the theoretical foundations of TIC in mental health care is crucial for several reasons. First, theory provides explanatory power by clarifying the mechanisms through which trauma impacts mental health and how TIC can mitigate these effects (Cloitre et al. 2011; Porges 2021). Second, grounding TIC in theory supports fidelity in implementation, ensuring that principles are consistently applied rather than selectively interpreted (Bryson et al. 2017). Third, theoretical clarity enhances the potential for interdisciplinary collaboration, allowing psychiatrists, nurses, psychologists, social workers, and peer providers to align around shared conceptual models (Isobel and Edwards 2017). Finally, identifying the dominant and emerging frameworks may guide future training, policy, and research priorities, supporting the continued transformation of mental health systems toward trauma‐informed and recovery‐oriented care (Slade et al. 2014). Given the conceptual heterogeneity of TIC in mental health, a scoping review provides a rigorous and appropriate methodology to map existing knowledge. Scoping reviews are designed to clarify concepts, examine the extent and nature of research activity, and identify gaps in the evidence base, particularly in areas where definitions are contested and the literature is multidisciplinary (Arksey and O'Malley 2005; Levac et al. 2010; Peters et al. 2020). Unlike systematic reviews, which address narrowly defined intervention questions, scoping reviews allow for broader exploration of theoretical and conceptual work. This is particularly relevant for TIC in mental health, where the literature includes empirical studies, theoretical articles, policy reports, and implementation guides. This scoping review therefore seeks to address the following research question: Which theoretical models or frameworks are used to understand trauma‐informed care in mental health settings? By mapping the theoretical foundations of TIC in mental health, this review will provide much‐needed conceptual clarity, highlight underexplored approaches, and inform both practice and policy. Ultimately, synthesizing theoretical models can strengthen the coherence and impact of TIC as a transformative framework for mental health care.

For mental health nursing, this theoretical clarity has direct practice relevance. Nurses frequently enact TIC through everyday relational encounters, trauma inquiry, de‐escalation, environmental safety, documentation, continuity of care, and advocacy around coercive practices. A nursing perspective therefore requires attention not only to what TIC principles are but to how different theories shape interpretations of distress, power, choice, safety, and therapeutic relationships (Isobel and Edwards 2017).

## 2 Methodology

### 2.1 Protocol and Registration

This scoping review is conducted in line with the PRISMA‐ScR (Preferred Reporting Items for Systematic Reviews and Meta‐Analyses Extension for Scoping Reviews) framework. The protocol was deposited in the Open Science Framework and remained unchanged over the course of the review.

### 2.2 Eligibility Criteria

We included peer‐reviewed journal articles published in English between 2010 and 2025. Studies were eligible if they examined TIC in mental health settings, involved service users or mental health professionals, and explicitly applied, discussed, or referenced one or more theoretical models or frameworks. Both empirical (qualitative, quantitative, or mixed methods) and theoretical or conceptual studies were included.

### 2.3 Information Sources and Search Strategy

We conducted a search in CINAHL, Scopus, Web of Science, and PubMed, chosen for their coverage of disciplines relevant to TIC, including nursing, psychiatry, psychology, social work, and health services. Backward citation tracking was used to identify additional studies. Grey literature was excluded to maintain theoretical and academic rigor. Search strategies combined controlled vocabulary (e.g., MeSH terms) and free‐text terms using Boolean operators, truncation, and phrase searching. For example, in PubMed we used: (“trauma‐informed care” OR “trauma informed”) AND (“mental health” OR psychiatry OR “mental health services”) AND (theor* OR framework* OR model*). All search strategies and dates were documented for transparency. All records were imported into a reference management system, and duplicates were removed. Screening followed PRISMA guidelines in two stages: title and abstract screening, followed by full‐text review by two independent reviewers. Articles were included if they met all criteria and explicitly linked TIC to a theoretical or conceptual framework.

### 2.4 Study Selection

**FIGURE 1:**
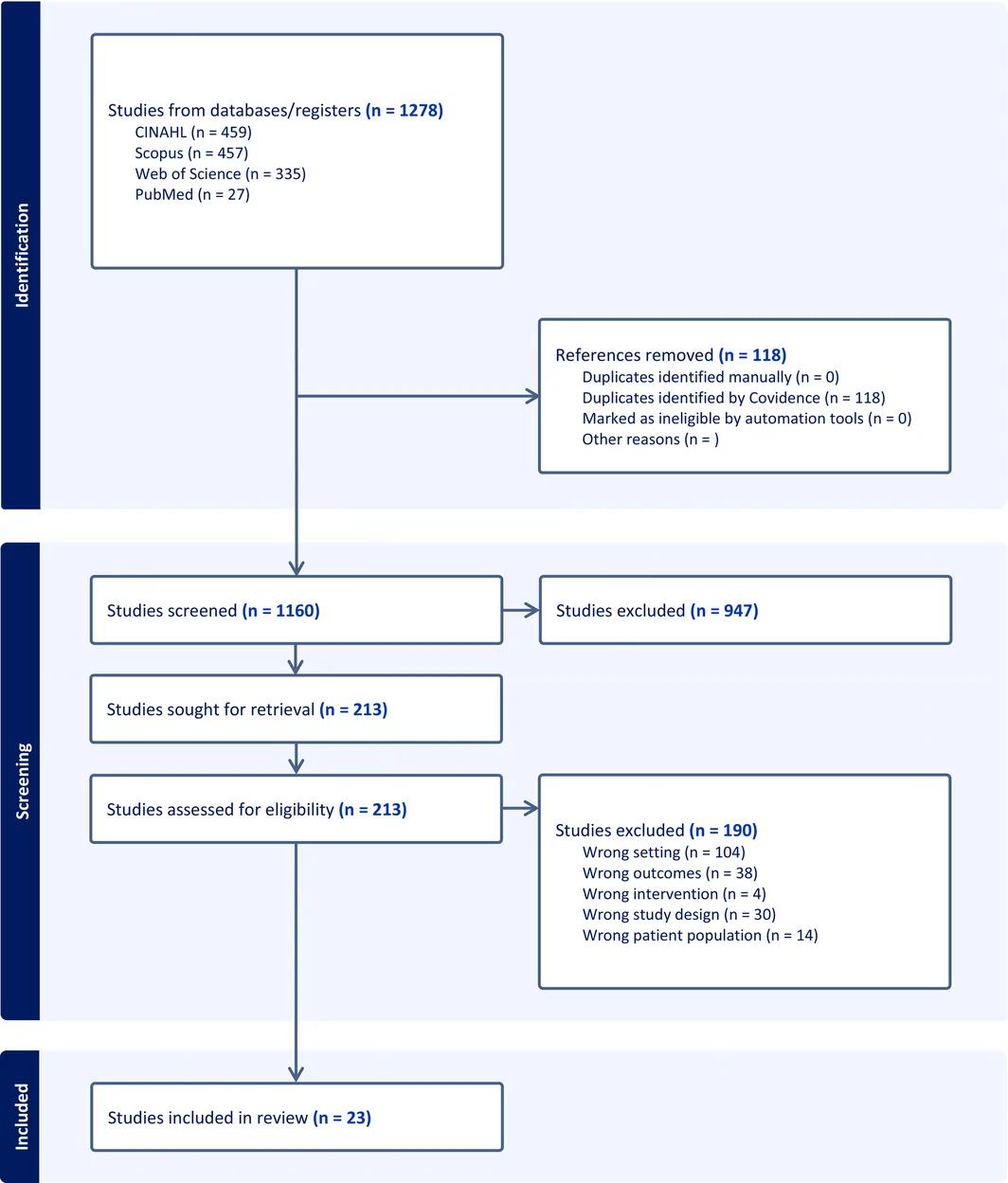
PRISMA flow diagram of study selection.

All identified studies were imported into Covidence and Mendeley. Duplicate records were removed using the Covidence review management platform (https://www.covidence.org). Two authors (K.J. and M.E.S.) independently screened the remaining titles and abstracts. Disagreements were resolved through discussion or consultation with a third reviewer, and the selection process was documented in a PRISMA flow diagram (Figure 1).

In scoping reviews, eligibility criteria do not always need to be fully defined prior to the literature search; instead, they may be refined as familiarity with the literature grows (Arksey and O'Malley 2005). Accordingly, the eligibility criteria in this review were adjusted following the initial title screening.

### 2.5 Data Extraction

Data extraction was carried out independently by two reviewers (K.J. and M.E.S.) using predefined and piloted extraction templates. Once data had been extracted, any discrepancies were discussed until consensus was reached. A third reviewer (M.H.M.) was available to support resolution if needed. For all included studies, data were systematically collected and recorded. This included information on authors, year and country of publication, study aim and design, context and population, theoretical or conceptual frameworks used, area of focus, as well as key findings and conclusions (Gill et al. 2014; Rogers et al. 2017).

All included studies were systematically reviewed, and data were charted according to predefined categories: author and publication details, study aim, context, focus area, theoretical content, key findings, and conclusions.

### 2.6 Data Synthesis

Data were synthesized using a qualitative, inductive approach consistent with Arksey and O'Malley's framework for scoping reviews (Arksey and O'Malley 2005), as refined by Levac et al. (2010) and guided by the JBI methodology. Because the purpose of this review was to map how theoretical frameworks inform TIC in mental health settings rather than assess intervention effectiveness or study quality, a thematic narrative synthesis was conducted. The synthesis followed the textual narrative approach described by Lucas et al. (2007), which supports structured comparison of study characteristics, theoretical content, and key findings across heterogeneous studies. The data synthesis proceeded through four iterative stages:

(1) Familiarization and coding of extracted data, focusing on explicit and implicit references to theoretical or conceptual frameworks. (2) Organization of codes into conceptual categories, including neurobiological, psychological, relational, socio‐ecological and organizational orientations. (3) Development of overarching themes, which resulted in the identification of seven theoretical domains of TIC. (4) Narrative integration, in which each domain was described in relation to how it conceptualizes trauma, informs clinical practice, guides organizational change, and relates to broader structural or systemic determinants.

Table 1 provides an illustrative example of how a meaning unit was progressively condensed, coded, categorized, and abstracted into an overarching theme.

**TABLE 1:** Example of the content‐analysis process.

Step	Analytic focus	Example content/action	Result
1. Raw data (meaning unit)	Identify a relevant segment from the included study	“The implementation process in Hawaiʻi was structured around SAMHSA's six trauma‐informed principles, requiring alignment of clinical practice, organizational policies, and leadership commitment” (Endres et al. 2015)	Meaning unit extracted
2. Condensation	Condense while preserving essential meaning	“SAMHSA principles guided practice, policy, and leadership alignment”	Condensed meaning unit
3. Initial codes	Generate descriptive labels	“SAMHSA principles,” “practice‐policy alignment,” “leadership commitment,” “system‐level implementation”	Codes created
4. Subcategory formation	Cluster related codes	“Organizational alignment with TIC principles”	Subcategory
5. Category formation	Abstract to a higher conceptual level	“System‐wide integration of trauma‐informed care”	Category
6. Thematic abstraction	Link category to overarching theme	Theme: “SAMHSA as a Cornerstone Framework for Trauma‐Informed Systems of Care”	Final theme
7. Analytic memo/rationale	Document interpretive reasoning	The study illustrates how SAMHSA's principles are operationalized not only in clinical interactions but also through leadership engagement and organizational restructuring. This justified defining SAMHSA as a central, cross‐cutting framework in the thematic synthesis	Reflexive analytic note

To enhance clarity, studies were initially grouped according to the perspective represented (e.g., service users, clinicians, organizational stakeholders). Patterns within and across these perspectives were then identified and summarized descriptively and narratively. Across the synthesis, particular attention was paid to the purpose of theory use, the degree of theoretical integration and areas of conceptual convergence, divergence and hybridization.

## 3 Result

### 3.1 Study Selection

A total of 1278 records were identified through database searches in CINAHL (*n* = 459), Scopus (*n* = 457), Web of Science (*n* = 335), and PubMed (*n* = 27). After removal of 118 duplicates, 1160 titles and abstracts were screened, resulting in the exclusion of 947 records. The remaining 213 full‐text articles were assessed for eligibility, of which 190 were excluded due to irrelevant population, setting, intervention, outcomes, or study design (Figure 1). In total, 23 studies met the inclusion criteria and were included in the final synthesis (Table 2).

**TABLE 2:** Selected articles.

Author(s), year of publication, and study location	Title	Study populations	Aims of the study	Methodology	Service user or/and health professional perspective	Important results
Alessi and Kahn (2019), USA	Using Psychodynamic Interventions to Engage in Trauma‐Informed Practice	Mental health practitioners and clients with complex trauma histories	To integrate psychodynamic principles into trauma‐informed practice for improved engagement and relational repair	Qualitative and theoretical synthesis drawing from clinical vignettes and psychodynamic theory	Health professional perspective	Demonstrates that psychodynamic understanding of transference and countertransference can deepen trauma‐informed relationships and reduce therapeutic ruptures
Endres et al. (2015), USA (Hawaiʻi)	Toward a Trauma‐Informed System of Care in Hawaiʻi's Adult Mental Health Division	State‐level administrators, providers, and consumers in Hawaiʻi's Adult Mental Health Division	To describe progress toward implementing a statewide trauma‐informed system of care in adult mental health services	Policy and system‐level descriptive report outlining SAMHSA TIC domains (safety, trust, choice, collaboration, empowerment)	System and organizational perspective	Reports statewide implementation progress; highlights success factors (training, policies, cross‐sector collaboration) and remaining barriers to full TIC integration
Epp et al. (2022), Canada	Maintaining Safety While Discussing Suicide: Trauma‐Informed Research in an Online Focus Group	Rural participants across six Canadian provinces (n not specified)	To explore trauma‐informed (TI) strategies for maintaining participant and researcher safety while discussing suicide in online focus groups	Qualitative design using online focus groups; thematic analysis supported by NVivo	Research participant and researcher perspectives	TI strategies (camera control, ongoing consent, localized support lists, and non‐judgmental facilitation) enhanced safety and openness; researcher debriefing mitigated vicarious distress. Challenges included limited observation of distress cues online
Finch et al. (2024), UK	Relationships Between Adverse Childhood Experiences, Attachment, Resilience, Psychological Distress and Trauma Among Forensic Mental Health Populations	128 forensic mental health service users in the UK	To explore how adverse childhood experiences (ACEs), attachment, and resilience predict psychological distress and trauma symptoms	Quantitative survey study using six validated instruments (ACE‐IQ, VASQ, CYRM, RRC‐ARM, CORE‐10, ITQ)	Service user perspective	High prevalence of ACEs and insecure attachment linked with low resilience and high trauma symptoms; resilience and attachment identified as intervention targets in forensic mental health
Gilbert et al. (2012), USA	Differential Service Utilization Associated With Trauma‐Informed Integrated Treatment for Women With Co‐occurring Disorders	Women with co‐occurring mental health and substance use disorders and trauma histories	To assess how trauma‐informed integrated treatment affects service utilization across sub‐groups of women	Quantitative secondary analysis of national study data; cluster analysis and 12‐month follow‐up	Service user perspective (program participants)	Integrated treatment improved service utilization and outcomes, especially among women with severe PTSD and substance abuse symptoms
Harris et al. (2025), UK	Comprehend, Cope and Connect: A Trauma‐Informed Holistic Approach to Address Mental Health Crisis as a Team	Staff teams and patients in acute and crisis mental health settings across England	To describe and advocate for the CCC (Comprehend, Cope and Connect) model as a national trauma‐informed intervention for crisis care	Conceptual and practice‐based article drawing on multi‐site implementation experiences	Both service user and health professional perspectives	CCC provides a shared trauma‐informed formulation framework enhancing team cohesion, coping, and patient engagement during crises
Isobel, Gladstone, et al. (2021), Australia and Canada	A Qualitative Inquiry into Psychiatrists' Perspectives on the Relationship of Psychological Trauma to Mental Illness and Treatment: Implications for Trauma‐Informed Care	Psychiatrists (n not specified)	To explore psychiatrists' understandings of trauma and implications for trauma‐informed care	Interpretive qualitative inquiry using semi‐structured interviews	Health professional perspective	Psychiatrists acknowledge trauma's role but vary in belief about causality; trauma‐informed care viewed as humanizing but not yet integrated into psychiatric models
Isobel, McCloughen, et al. (2021), Australia	Intergenerational Trauma and Its Relationship to Mental Health Care: A Qualitative Inquiry	13 psychiatrists working in public mental health services	To explore psychiatrists' understandings of intergenerational trauma and implications for trauma‐informed care	Qualitative study; semi‐structured interviews analysed thematically using Braun and Clarke (2006)	Health professional perspective	Four themes identified—awareness, powerlessness, thinking differently, and looking backward/forward. Psychiatrists recognized trauma transmission but felt constrained by systemic barriers; calls for training and interprofessional collaboration
Jacobowitz et al. (2015), USA	Post‐Traumatic Stress, Trauma‐Informed Care, and Compassion Fatigue in Psychiatric Hospital Staff: A Correlational Study	172 psychiatric hospital staff (RNs, aides, assistant counsellors, psychiatrists, case coordinators, and rehabilitation specialists) in a 150‐bed New York psychiatric hospital	To identify factors associated with post‐traumatic stress symptoms (PTSS) among inpatient psychiatric staff and explore the role of trauma‐informed care (TIC) and compassion fatigue	Quantitative, descriptive correlational design using validated instruments: PCL‐C (PTSD symptoms), RS‐14 (resilience), ProQOL (Compassion Satisfaction & Burnout), CCPAI (confidence in coping with aggression), and LEC (non‐work trauma); hierarchical regression analysis	Health professional perspective	Frequent exposure to verbal and physical assaults; mean = 4 verbal assaults/month, 1 physical attack/month. Significant predictors of PTSS were (1) longer time since last trauma‐informed care meeting and (2) higher burnout. Age and compassion satisfaction also contributed. Findings highlight importance of regular TIC training and burnout prevention to reduce staff trauma symptoms
Lietz et al. (2014), USA	A Case Study Approach to Mental Health Recovery: Understanding the Importance of Trauma‐Informed Care	One woman with previous diagnosis of severe mental illness	To explore how a person with severe mental illness progresses to functional recovery and the role of trauma‐informed care	Qualitative case study; semi‐structured interview triangulated with medical records; NVivo‐assisted analysis	Service user perspective	Recovery is possible; trauma‐informed and supportive relationships facilitate recovery; highlights need to view recovery beyond medication compliance
Mihelicova et al. (2018), USA	Trauma‐Informed Care for Individuals with Serious Mental Illness: An Avenue for Community Psychology's Involvement in Community Mental Health	Individuals with serious mental illness receiving community mental health services	To explore the role of community psychology in advancing trauma‐informed care for individuals with serious mental illness	Conceptual review linking trauma‐informed care with community psychology principles (ecological models, empowerment)	Health professional and system‐level perspectives	Advocates for community psychologists to enhance TIC implementation via ecological frameworks, empowerment, and system‐level consultation
Mirick et al. (2022), USA	Trauma‐Informed Clinical Practice with Clients with Suicidal Thoughts and Behaviors	Clients experiencing suicidal ideation and mental health practitioners	To describe a trauma‐informed approach to working with clients who experience suicidal thoughts and behaviors	Conceptual and clinical case‐based paper grounded in evidence‐informed trauma and suicide prevention literature	Health professional perspective	Highlights importance of understanding suicidality within the context of trauma; emphasizes collaborative, empathic, and safety‐oriented practice to avoid retraumatization
Palfrey et al. (2019), Australia	Achieving Service Change Through the Implementation of a Trauma‐Informed Care Training Program Within a Mental Health Service	Nursing, medical, and allied health professionals in mental health settings	To evaluate the impact of a one‐day trauma‐informed care (TIC) training workshop on clinicians' awareness, attitudes, and practices	Quantitative pre/post design with follow‐up survey; statistical comparison (*p* < 0.001)	Health professional perspective	TIC training significantly improved confidence and awareness, reduced perceived barriers, and increased routine trauma screening and intervention use
Price et al. (2024), UK	De‐escalating Aggression in Acute Inpatient Mental Health Settings: A Behavior Change Theory‐Informed, Secondary Qualitative Analysis of Staff and Patient Perspectives	Ward staff (*n* = 20) and patients (*n* = 26) from acute and psychiatric intensive care settings	To identify behavioral drivers influencing effective de‐escalation practices in mental health wards	Secondary qualitative analysis using Theoretical Domains Framework (TDF)	Both service user and health professional perspectives	Effective de‐escalation depends on trauma awareness, emotional regulation, relational safety, and supportive organizational culture
Rienecke et al. (2021), USA	Posttraumatic Stress Disorder Symptoms and Trauma‐Informed Care in Higher Levels of Care for Eating Disorders	613 adults with eating disorders in inpatient, residential, and partial hospitalization care	To examine prevalence and changes in PTSD symptoms in eating disorder treatment programs with trauma‐informed components	Quantitative pre‐post design using PTSD Checklist‐5 (PCL‐5) at admission and discharge	Service user perspective	Over half showed PTSD symptoms at admission; PTSD scores decreased significantly during treatment even without formal trauma therapy, supporting benefits of trauma‐informed approaches
Rose et al. (2012), UK	Despite the Evidence—Why Are We Still Not Creating More Trauma‐Informed Mental Health Services?	Policy and literature sources related to UK mental health services	To examine why trauma‐informed practices have not been fully implemented despite strong evidence and policy recommendations	Policy and literature analysis	System‐level/policy perspective	Implementation of trauma‐informed care remains patchy despite evidence; calls for systemic policy change and leadership support
Scoglio et al. (2020), USA	Trauma‐Informed Drug Screens for Veterans with Co‐occurring Disorders: A Case Series	Three male veterans with PTSD and substance use disorders in outpatient addiction recovery programs	To describe implementation of a trauma‐informed approach (GLAPE) to urine drug screening for veterans with co‐occurring disorders	Qualitative case series with descriptive analysis of clinical implementation	Health professional and service user perspectives	GLAPE approach (Guidance, Listening, Articulating options, Permission, Evaluation) improved engagement and retention by reducing retraumatization and promoting collaborative monitoring
Shier and Turpin (2022), Canada	Trauma‐Informed Organizational Dynamics and Client Outcomes in Concurrent Disorder Treatment	172 service users in concurrent disorder treatment programs	To test how trauma‐informed organizational environments predict client intrapersonal outcomes and reduction in concurrent disorder behaviors	Cross‐sectional quantitative survey; multivariate analysis validating trauma‐informed organizational model (safety, trust, choice, collaboration, empowerment)	Service user perspective	Trauma‐informed environments significantly predict improved self‐awareness, coping, and reduction in disorder‐related behaviors; supports organizational‐level TIC implementation
Thordarson and Rector (2020), USA	From Trauma‐Blind to Trauma‐Informed: Re‐thinking Criminalization and the Role of Trauma in Persons with Serious Mental Illness	People with serious mental illness (SMI) in forensic and community mental health systems	To examine how trauma contributes to criminalization of persons with SMI and argue for trauma‐informed systems	Conceptual review and synthesis of evidence	System‐level/health professional perspective	Trauma‐related behaviors are often misinterpreted; trauma‐informed approaches reduce criminalization and improve safety and outcomes
Voith et al. (2020), USA	Using a Trauma‐Informed, Socially Just Research Framework with Marginalized Populations: Practices and Barriers to Implementation	The study focuses on low‐income African American men involved in the criminal justice system, not Native American or immigrant groups	To present a framework for conducting socially just, trauma‐informed research addressing power and safety for marginalized groups	Qualitative participatory research framework based on community‐based participatory research principles	Researcher and community participant perspectives	Identifies barriers such as institutional power hierarchies and researcher positionality; emphasizes trust, cultural humility, and shared decision‐making as trauma‐informed principles
Wilson et al. (2023), Australia & New Zealand	Promoting Mental Health Recovery by Design: Physical, Procedural, and Relational Security in the Context of the Mental Health Built Environment	38 participants including inpatients, carers, and staff in an Australian regional mental health unit	To explore how design elements of mental health units can promote recovery and trauma‐informed safety	Qualitative descriptive study using focus groups and interviews with patients, carers, and professionals	Both service user and health professional perspectives	Identifies three dimensions of security (physical, procedural, relational); recommends trauma‐informed design to enhance safety, dignity, and recovery orientation
Wu et al. (2020), Hong Kong, China	Patients' Reports of Traumatic Experience and Posttraumatic Stress in Psychiatric Settings	129 psychiatric inpatients in Hong Kong	To determine prevalence and effects of traumatic experiences (TE) in psychiatric settings and their association with distress, anxiety, and depression	Quantitative survey using Life Event Checklist (LEC), Psychiatric Experiences Questionnaire (PEQ), and Impact of Event Scale‐Revised (IES‐R)	Service user perspective	84.5% experienced trauma in psychiatric settings, including restraint and witnessing aggression; TE associated with high distress and supports need for trauma‐informed psychiatric care
Xiao et al. (2016), Australia	Do Mental Health Clinicians Elicit a History of Previous Trauma in Female Psychiatric Inpatients?	100 female psychiatric inpatients in Melbourne, Australia	To determine whether clinicians routinely obtain trauma histories during inpatient admissions	Retrospective file audit of 100 patient records	Health professional and documentation audit perspective	Over half of records lacked trauma documentation; highlights need for mandatory trauma inquiry and staff training in trauma‐informed care

### 3.2 Description of Included Studies

The 23 included studies were published between 2010 and 2025 and originated from a range of high‐income countries. The largest number came from the United States, followed by Australia, the United Kingdom, Canada, and New Zealand, with additional contributions from European and Asian contexts. Most studies were qualitative, while a smaller number used mixed‐methods or quantitative designs. Data collection methods commonly included semi‐structured interviews, focus group interviews, observations, clinical case material, or document analysis. Mixed‐methods studies additionally incorporated questionnaires and administrative data. The most frequently used analytical approaches were thematic analysis and content analysis, while a few studies applied grounded theory or conceptual/theoretical synthesis. Across the studies, there was considerable variation in the populations represented, including service users with complex trauma histories, mental health practitioners (e.g., nurses, psychiatrists, psychologists), organizational leaders or policymakers, and in some cases, both service user and provider perspectives. Most studies explored trauma‐informed care in adult mental health services, with settings spanning inpatient units, community care, forensic services, and organizational or system‐level perspectives. The majority of studies adopted a trauma‐informed lens but varied in the degree to which theoretical frameworks were explicitly articulated. Some were grounded in established models such as SAMHSA's trauma‐informed care principles, attachment theory, or polyvagal theory, while others used multiple or hybrid frameworks, or drew implicitly on trauma concepts without naming a specific theoretical model. Sample sizes ranged widely, from single‐case studies to large survey‐based designs with over 600 participants. Most qualitative studies used purposive sampling, whereas quantitative designs were more likely to employ convenience or random sampling strategies. Table 2 presents a detailed overview of the characteristics of the included studies. Across the 23 included studies, seven overarching thematic domains were identified: (1) neurobiological and psychophysiological foundations, (2) psychological and relational models, (3) ACEs and social determinants frameworks, (4) SAMHSA as a cornerstone framework for trauma‐informed systems of care, (5) socio‐ecological and intersectional frameworks, (6) organizational and implementation science models, and (7) integrated or hybrid approaches. Collectively, these themes illustrate the multidimensional nature of trauma and the evolving field of TIC, offering valuable insights for theoretical advancement, practical implementation, and future research.

#### 3.2.1 Theme 1: Neurobiological and Psychophysiological Foundations

A prominent theoretical strand in the TIC literature situates trauma primarily as a neurobiological and psychophysiological phenomenon, highlighting how traumatic experiences dysregulate stress response systems, particularly the hypothalamic–pituitary–adrenal (HPA) axis, autonomic nervous system, and neurocognitive processing. This perspective draws heavily on emerging evidence in Polyvagal theory and stress physiology, positioning trauma not merely as a psychological event but as an embodied experience that fundamentally alters neurobiological functioning (Porges 2021). From this standpoint, trauma‐related behaviors such as hyperarousal, dissociation, avoidance, and heightened threat perception are understood as adaptive survival responses rather than symptoms of pathology.

This theoretical grounding is reflected across several studies that emphasize how safety, de‐escalation, and trauma‐sensitive care must be structured around an awareness of how trauma shapes perception, stress reactivity, and sense of safety. For example, (Wilson et al. 2023) demonstrated how the physical, procedural, and relational dimensions of psychiatric unit design can support or undermine trauma recovery. They argue that environments must be explicitly designed to minimize sensory triggers, enhance predictability, and build relational trust, thereby aligning architectural and procedural choices with neurobiological understandings of safety. This aligns with TIC's core principle of “safety first” both physical and psychological, recognizing that dysregulated stress systems require stabilizing environments to enable therapeutic engagement.

Similarly, (Wu et al. 2020) provided empirical evidence of the high prevalence of trauma and post‐traumatic stress linked to coercive experiences in psychiatric inpatient care. Their study found that 84.5% of patients reported at least one traumatic event during admission, with frequent exposure to restraint and aggression. These findings make explicit that mental health inpatient settings can themselves be sources of trauma when care involves coercion, restraint, aggression, loss of control, or other experiences of threat. A trauma‐informed response therefore requires practices that minimize coercion, recognize the impact of the care environment itself, and prioritize safety, trust, and choice.

Across the literature, heightened physiological arousal and threat sensitivity are shown to impact both patients and staff. A study found that psychiatric staff frequently experienced verbal and physical assaults, with associated post‐traumatic stress symptoms strongly linked to burnout and reduced engagement with trauma‐informed care (Jacobowitz et al. 2015). This illustrates that neurobiological stress mechanisms operate on both sides of the care relationship. When staff themselves are dysregulated, their capacity to provide safe, attuned, and trauma‐sensitive care diminishes, creating a feedback loop that can perpetuate distress and conflict in psychiatric wards. The physiological understanding of trauma also shapes clinical practice strategies. Price et al. (2024) highlighted that effective de‐escalation in acute inpatient settings relies on trauma awareness and emotion regulation, both for staff and service users (Price et al. 2024). Within biomedical or risk‐dominant ward cultures, expressions of distress or resistance may be interpreted as pathology or aggression and can become further justification for coercive responses. A trauma‐informed interpretation instead considers how behavior may communicate threat, distress, or attempts to regain safety and control; this supports grounding, collaborative de‐escalation, and the least coercive response possible. This resonates with findings from (Palfrey et al. 2019), who showed that TIC training significantly improved clinicians' confidence and reduced perceived barriers, indicating a shift toward relational and physiological safety in clinical encounters.

Other studies extend this neurobiological perspective to treatment outcomes. Rienecke et al. (2021) demonstrated that trauma‐informed approaches in eating disorder treatment were associated with significant reductions in PTSD symptom scores during admission. Notably, these improvements occurred even without formal trauma therapy, suggesting that creating a predictable, non‐threatening, and safety‐oriented environment may directly influence neurobiological regulation and support stabilization (Rienecke et al. 2021).

Furthermore, Mirick et al. (2022) emphasized the relevance of trauma‐informed approaches for clients experiencing suicidality. They argue that trauma responses often underpin acute crises and that collaborative, empathic, and non‐pathologizing clinical practices can counteract the sense of threat and disconnection that fuels suicidal ideation. This perspective aligns closely with polyvagal theory's focus on co‐regulation, emphasizing how safe and attuned interpersonal interactions can help restore physiological regulation (Mirick et al. 2022). Finally, from a neurobiological lens, screening and inquiry practices also come into focus. Xiao et al. (2016) documented widespread gaps in trauma history documentation during psychiatric admissions, reflecting a persistent disconnect between neurobiological theory and clinical routines. Trauma is often unassessed and unaddressed, despite its known physiological and psychological implications. Bridging this gap requires not only staff training but systemic changes in assessment protocols to ensure that neurobiological insights inform everyday clinical practice (Xiao et al. 2016).

#### 3.2.2 Theme 2: Psychological and Relational Models

A second major theme emphasizes that trauma is not only a neurobiological phenomenon but also fundamentally a relational rupture that disrupts trust, safety, and the capacity to connect with others. Trauma‐informed care (TIC) is therefore often conceptualized as relational practice, focusing on the therapeutic alliance, empathic attunement, and collaborative meaning‐making between service users and professionals. This theme draws heavily on attachment theory, psychodynamic perspectives, and other relational frameworks to illuminate the interpersonal dimensions of trauma recovery.

Attachment theory highlights how early adverse experiences shape internal working models of self and others, often resulting in insecure or disorganized attachment patterns that affect how individuals perceive safety, trust, and intimacy later in life. These disruptions are directly relevant to mental health settings, where service users may re‐experience dynamics of threat or abandonment within therapeutic relationships. Relational safety, a sense that the interpersonal environment is predictable, respectful, and attuned, is therefore critical to effective trauma‐informed practice. As Harris et al. (2025) argue, trauma‐informed interventions must establish a shared trauma‐informed formulation framework enhancing team cohesion, coping, and patient engagement, recognizing that relational dynamics shape both individual recovery trajectories and organizational culture.

Psychodynamic frameworks further deepen this understanding by addressing the unconscious relational patterns that often emerge in therapeutic contexts. As Alessi and Kahn (2019) observe, psychodynamic interventions deepen trauma‐informed engagement by addressing transference and relational ruptures (Alessi and Kahn 2019). Trauma survivors may unconsciously re‐enact early experiences of powerlessness, betrayal, or abandonment, and staff may similarly react defensively or withdraw. By naming and working through these dynamics, psychodynamic TIC offers a pathway to repair trust, sustain engagement, and strengthen therapeutic alliances.

Several empirical studies support these relational insights. Isobel, Gladstone, et al. (2021) show how psychiatrists recognize the role of trauma in shaping patient engagement and intergenerational transmission, even as they navigate systemic constraints that hinder the integration of trauma‐informed principles into psychiatric care. Their findings underscore how professionals often acknowledge the relational impact of trauma but require organizational and cultural support to act on this awareness (Isobel, Gladstone, et al. 2021; Isobel, McCloughen, et al. 2021). Similarly, Jacobowitz et al. (2015) highlight how staff in psychiatric settings are themselves vulnerable to trauma exposure, leading to compassion fatigue and burnout both of which can undermine relational safety unless trauma‐informed frameworks are consistently embedded into team practices (Jacobowitz et al. 2015).

Relational models are also evident in clinical training and service development. Palfrey et al. (2019) demonstrate how targeted TIC training improves clinicians' confidence, awareness, and ability to engage patients collaboratively (Palfrey et al. 2019), while Price et al. (2024) illustrate that effective de‐escalation in acute psychiatric settings depends on emotional regulation, empathy, and organizational support that prioritize relational safety. Scoglio et al. (2020) further show how even procedural interventions such as drug screening can be reshaped through collaborative, permission‐based dialogues that minimize traumatization and enhance trust (Scoglio et al. 2020).

From the service user perspective, Lietz et al. (2014) highlight how recovery from severe mental illness was experienced as relational and grounded in trauma‐informed, supportive relationships—not just medical interventions. Their qualitative case study reflects how trust and safety, rather than coercion or clinical detachment, foster meaningful recovery. These findings align with a growing body of literature that positions relationships as the vehicle of change, rather than a backdrop to treatment (Lietz et al. 2014).

Taken together, these studies reveal that TIC is inseparable from relational processes. Attachment‐informed and psychodynamic approaches help practitioners understand how trauma disrupts trust and connection, while practical TIC frameworks translate this insight into organizational and clinical practices that foster safety, empathy, and collaboration. Importantly, these models also acknowledge staff vulnerability both to their own trauma histories and to the impact of working in high‐intensity settings, highlighting the need for trauma‐informed organizational cultures, reflective practice, and mutual support structures.

#### 3.2.3 Theme 3: ACEs and Social Determinants Frameworks

A large subset of studies employs ACEs frameworks and social determinants of health perspectives to conceptualize trauma not as an isolated event but as systemic, pervasive, and cumulative. This framing challenges traditional psychiatric models that often treat trauma as an “add‐on” or secondary factor. Instead, these studies argue that trauma exposure represents a baseline reality for many mental health populations—a reality that necessitates universal, trauma‐informed responses across care systems rather than selective or exceptional approaches.

Such an orientation reflects a population‐level lens on trauma. Rather than focusing solely on individual pathology, ACE frameworks highlight how cumulative exposure to early adversity such as abuse, neglect, community violence, or systemic discrimination profoundly shapes developmental trajectories, attachment patterns, stress physiology, and later mental health outcomes. For example, Finch et al. (2024) found that “insecure attachment and high ACE exposure predicted higher trauma symptoms and lower resilience” (3), identifying early adversity as a powerful predictor of psychological distress in forensic mental health populations. This reinforces the argument that TIC must be designed as a preventive, population‐based intervention rather than a specialized clinical program for a few (Finch et al. 2024).

The pervasiveness of trauma exposure within psychiatric settings themselves further supports this position. Wu et al. (2020) documented that 84.5% of psychiatric inpatients reported traumatic experiences during hospitalization, including coercive measures and witnessing aggression. These findings illustrate how trauma is both a precursor to and a product of mental health care systems, creating a cycle of vulnerability and retraumatization if not adequately addressed (Wu et al. 2020). Similarly, Xiao et al. (2016) revealed that over half of psychiatric inpatient records lacked documented trauma histories, underscoring a significant implementation gap between recognition of trauma prevalence and actual clinical practice (Xiao et al. 2016).

This gap between trauma prevalence and actual clinical practice has also been highlighted in previous conceptual and empirical work. Gilbert et al. (2012) demonstrated that trauma‐informed integrated treatment improved service utilization and outcomes for women with co‐occurring mental health and substance‐use conditions (Gilbert et al. 2012), while Lietz et al. (2014) underscored the centrality of supportive, trauma‐informed relationships in recovery trajectories. Similarly, Jacobowitz et al. (2015) highlighted the impact of trauma exposure on psychiatric staff, linking lack of TIC structures to increased post‐traumatic stress symptoms and burnout indicating that systemic trauma frameworks must address both patient and staff well‐being (Jacobowitz et al. 2015).

Framed through ACE and social determinant models, trauma becomes a structural and relational phenomenon, not just an individual clinical variable. This has critical implications for practice. First, it justifies universal trauma screening and routine trauma inquiry rather than selective assessments based on clinical suspicion. Second, it calls for trauma‐informed strategies to be embedded in organizational culture, design, and everyday interactions, what some authors describe as “universal precautions” in mental health care. Third, it places emphasis on upstream, preventive interventions, strengthening resilience and protective factors before acute crises occur.

Finally, this framing provokes important reflections on power, equity, and responsibility in mental health systems. By foregrounding trauma as systemic, these frameworks make visible how structural inequities such as poverty, racism, gender‐based violence, and coercive psychiatric practices are not peripheral issues but core determinants of mental health. Studies like Thordarson and Rector (2020) and Rose et al. (2012) call for system‐level transformation, not just clinical adaptation. They argue that without explicit attention to trauma's systemic roots, TIC risks becoming a rhetorical or superficial practice, rather than a meaningful shift toward equity and healing (Rose et al. 2012; Thordarson and Rector 2020).

Conceptually, Theme 3 is distinguished from Theme 5 by its primary analytic level. Theme 3 groups frameworks that map cumulative exposure to adversity across the life course and the population distribution of social determinants such as poverty, violence, and discrimination. Theme 5, by contrast, focuses on how intersecting identities, power relations, colonization, racism, gender, and institutional structures shape both exposure to trauma and the meaning and consequences of that trauma across individual, community, organizational, and policy levels. The domains overlap, but they ask different theoretical questions: Theme 3 foregrounds cumulative adversity and its determinants, whereas Theme 5 foregrounds power, positionality, culture, and multi‐level context.

#### 3.2.4 Theme 4: SAMHSA—A Cornerstone Framework for Trauma‐Informed Systems of Care

The most consistently cited and operationalized framework across the included literature is the Substance Abuse and Mental Health Services Administration TIC model, frequently paired with the seminal conceptualization developed by Endres et al. (2015). Together, these frameworks articulate TIC through six interrelated principles: safety; trustworthiness and transparency; peer support; collaboration and mutuality; empowerment; and cultural, historical, and gender responsiveness. These principles provide not only a philosophical orientation but also a practical structure for transforming mental health services into environments that actively resist traumatization and foster recovery. A key strength of the framework lies in its explicit alignment between individual clinical encounters and broader organizational culture and system structures (Shier and Turpin 2022). As Endres et al. (2015) demonstrate in their statewide implementation in Hawaiʻi, a trauma‐informed system of care requires structural, policy, and cultural alignment with trauma‐informed principles (Endres et al. 2015). This articulation moves TIC beyond the realm of clinical interaction into the sphere of governance, workforce training, and policy design. Their experience also underscores that sustainable implementation demands multilevel strategies involving leadership commitment, cross‐sector collaboration, and the establishment of supportive infrastructures such as TIC champions and interdisciplinary workgroups. Other studies similarly underscore the operational impact of this framework. For example, Palfrey et al. (2019) report that structured TIC training based on SAMHSA principles significantly improved clinicians' confidence, increased routine trauma screening, and reduced perceived implementation barriers (Palfrey et al. 2019). This suggests that the model functions not only as a theoretical scaffold but also as a change lever within complex healthcare systems. Likewise, Shier and Turpin (2022) provide quantitative evidence that trauma‐informed organizational environments explicitly structured around safety, trust, collaboration, and empowerment are positively associated with improved intrapersonal coping and reductions in distress‐related and coping responses among people with concurrent mental health and substance‐use needs, strengthening the case for system‐level integration of TIC principles (Shier and Turpin 2022). Importantly, several studies illustrate that embedding these principles into everyday practice requires more than a checklist approach. Jacobowitz et al. (2015) highlight how infrequent TIC meetings and inadequate burnout prevention strategies are significantly associated with increased trauma symptoms among psychiatric staff. Their findings reflect the reciprocal nature of trauma‐informed environments: when organizational structures neglect staff well‐being, the capacity to deliver trauma‐informed care to service users is compromised (Jacobowitz et al. 2015). Similarly, Scoglio et al. (2020) show how the GLAPE trauma‐informed approach to drug screening enhanced engagement and reduced retraumatization among veterans, reinforcing the adaptability of SAMHSA's principles across diverse clinical settings (Scoglio et al. 2020). The literature also reveals critical gaps and implementation challenges that require reflective consideration. Xiao et al. (2016) found that more than half of inpatient psychiatric records lacked documentation of trauma histories, signalling persistent gaps between formal frameworks and everyday clinical practice. This suggests that although SAMHSA's model provides a shared language, its operationalization is uneven and may be undermined by structural barriers such as time pressures, documentation practices, and insufficient training. Thordarson and Rector (2020) extend this critique to the forensic context, arguing that responses to trauma are often misinterpreted, contributing to criminalization rather than recovery‐oriented support—an example of structural misalignment that TIC seeks to address but has not yet fully overcome (Thordarson and Rector 2020). Collectively, these findings reveal both the strength and vulnerability of relying on SAMHSA as a cornerstone framework for trauma‐informed care. Its widespread adoption reflects its conceptual clarity, scalability, and ability to bridge clinical practice, organizational culture, and policy domains. However, the variability in how its principles are applied, ranging from comprehensive system redesign (Endres et al. 2015) to partial or inconsistent implementation (Xiao et al. 2016), highlights the importance of sustained organizational commitment and reflexive practice. Effective implementation therefore requires more than adopting principles; it demands cultivating trauma‐informed cultures grounded in continuous learning, critical reflection, and shared responsibility.

#### 3.2.5 Theme 5: Socio‐Ecological and Intersectional Frameworks

A significant body of literature highlights the importance of socio‐ecological and intersectionality frameworks in understanding trauma not merely as an individual psychological event, but as a structural and relational phenomenon shaped by broader social forces. These perspectives extend the conceptual scope of trauma‐informed care by linking individual trauma experiences to systemic inequities, power dynamics, and structural determinants such as racism, colonization, poverty, and marginalization. This framing situates trauma within multi‐layered contexts from the individual and relational, to community, organizational, and policy levels underscoring that meaningful interventions must address not only clinical practice but also structural and institutional change. Several studies explicitly foreground this broader lens. For example, Voith et al. (2020) argue that intersectionality becomes the lens through which every other trauma‐informed principle is implemented (Voith et al. 2020). By centring intersectionality, their work emphasizes cultural humility, the need to share power between professionals and communities, and the necessity of creating safe spaces where trauma and systemic oppression can be named and addressed. Similarly, Thordarson and Rector (2020) critique the criminalization of trauma survivors, noting how trauma‐related behaviors among people experiencing significant mental health difficulties are often misinterpreted through a punitive lens, reflecting systemic neglect of trauma‐informed systems. Their conceptual analysis calls for policy and structural reforms that embed trauma‐awareness into the justice and mental health interface, rather than perpetuating cycles of surveillance and punishment (Thordarson and Rector 2020). These frameworks move beyond clinical encounters to highlight how trauma is distributed unevenly across populations due to historical and structural forces. Endres et al. (2015) describe statewide trauma‐informed implementation in Hawaiʻi, which was explicitly designed to address structural inequities and promote cross‐sector collaboration (Endres et al. 2015). Epp et al. (2022) show how trauma‐informed research practices can enhance safety for marginalized participants, foregrounding localized supports, ongoing consent, and power‐sensitive facilitation strategies (Epp et al. 2022). Isobel, McCloughen, et al. (2021) further underscore the significance of intergenerational trauma and systemic barriers to care, illustrating how structural determinants can shape both mental health trajectories and access to services (Isobel, McCloughen, et al. 2021).

Taken together, these studies position intersectionality and socio‐ecological perspectives as critical theoretical anchors for trauma‐informed care. They challenge service systems to look upstream to the social and policy environments that create and perpetuate trauma while also looking inward to transform practices, power relations, and organizational cultures. Rather than framing trauma solely as an individual pathology, these frameworks demand a multi‐level intervention logic, one that includes culturally responsive clinical care, trauma‐informed organizational change, cross‐sector collaboration, and policy advocacy for structural justice. They also call attention to the ethical imperative of shared power and community participation, aligning with broader movements toward social justice and equity in mental health.

Cultural, historical, and intergenerational dimensions are therefore integral to this domain rather than optional additions to TIC. At the same time, the included evidence only partially addresses these dimensions. Indigenous peoples, refugees and asylum seekers, and people exposed to racism and discrimination are not substantively represented as distinct populations in the included studies. This absence should be understood as a gap in the mapped evidence base, particularly because intersectional and socio‐ecological frameworks require attention to historical violence, displacement, racism, and culturally situated understandings of safety and healing.

#### 3.2.6 Theme 6: Organizational and Implementation Science Frameworks

Many studies adopted organizational change and implementation science frameworks to conceptualize TIC as a system‐level transformation rather than a discrete intervention. This reflects a shift from individual clinician competencies to organizational cultures and infrastructures capable of sustaining trauma‐aware practice over time. These frameworks emphasize leadership engagement, workforce development, and structural changes such as policy frameworks, environmental design, and service‐user involvement as essential conditions for embedding TIC (Endres et al. 2015; Rose et al. 2012; Wilson et al. 2023). A central insight across the literature is that implementation is most effective when TIC is framed not as an “add‐on” but as an overarching organizational philosophy shaping everyday interactions, routines, and accountability mechanisms (Scoglio et al. 2020; Shier and Turpin 2022). For example, Palfrey et al. (2019) demonstrated that even short, structured training interventions significantly improved clinician confidence in trauma inquiry and routine screening, signalling how targeted educational strategies can catalyses broader organizational change (Palfrey et al. 2019). Similarly, Shier and Turpin (2022) showed that trauma‐informed organizational environments predicted improved self‐awareness, enhanced coping, and reduced distress‐related and coping responses among people with concurrent mental health and substance‐use needs, underscoring the relational and cultural dimensions of TIC implementation. These findings align with implementation science principles, which emphasize contextual fit, staff engagement, and adaptive leadership as critical drivers of sustainable change (Shier and Turpin 2022). Several studies provide empirical evidence of organizational‐level strategies that operationalize these frameworks. Endres et al. (2015) described a statewide policy implementation in Hawaiʻi's adult mental health services that prioritized cross‐sector collaboration, policy alignment, and workforce training, identifying both enablers and persistent systemic barriers (Endres et al. 2015). Rose et al. (2012) highlighted why, despite strong evidence and policy recommendations, TIC implementation often remains patchy citing lack of sustained leadership commitment and structural integration as key obstacles (Rose et al. 2012). Wilson et al. (2023) expanded this understanding by showing how the built environment itself can either reinforce or undermine trauma‐informed principles, pointing to the role of physical, procedural, and relational security in supporting mental health recovery (Wilson et al. 2023). Further, studies like Scoglio et al. (2020) illustrated how trauma‐informed approaches embedded in routine procedures such as drug screening for veterans can transform traditionally retraumatizing practices into collaborative and empowering interactions, emphasizing the importance of procedural redesign (Scoglio et al. 2020). Xiao et al. (2016) identified significant gaps in routine trauma inquiry, suggesting that without clear institutional policies and accountability structures, TIC principles may remain aspirational rather than enacted in daily practice (Xiao et al. 2016). Collectively, these findings reflect a growing consensus that TIC implementation is not merely a matter of individual skill acquisition but a structural and cultural transformation. This aligns with broader implementation science frameworks that stress the importance of leadership, infrastructure, and continuous feedback loops in sustaining change. It also resonates with organizational learning theories, suggesting that creating trauma‐informed systems requires both top‐down strategic vision and bottom‐up practice engagement. As a result, successful TIC integration depends on organizations' ability to balance formal structures (e.g., policies, protocols, design) with informal processes (e.g., culture, values, relational practices) to create environments that consistently embody trauma‐informed principles at all levels of care delivery.

#### 3.2.7 Theme 7: Integrated and Hybrid Models

Most studies in the review adopt hybrid approaches, combining explanatory models (e.g., ACEs, neurobiological frameworks, attachment theory, psychodynamic and ecological perspectives) with implementation models (e.g., Substance Abuse and Mental Health Services Administration trauma‐informed care domains, organizational change frameworks, and service design approaches). This pragmatic blending reflects the complex realities of mental health practice, where trauma manifests simultaneously at individual, interpersonal, and systemic levels. Rather than committing to a single conceptual model, many authors strategically combine multiple frameworks to address both mechanisms of trauma impact and practical strategies for system change. For example, Harris et al. (2025) integrate a trauma formulation model with team‐based crisis care through the Comprehend, Cope, Connect (CCC) approach. This model does not rest solely on neurobiological or psychological explanations; rather, it operationalizes trauma theory into concrete, collaborative crisis responses that structure staff teamwork and patient engagement (Harris et al. 2025). Similarly, Scoglio et al. (2020) blend the Substance Abuse and Mental Health Services Administration trauma‐informed principles with addiction treatment models through the GLAPE framework, illustrating how trauma concepts can be adapted to specific practice contexts to minimize retraumatization and enhance engagement (Scoglio et al. 2020). A reflective reading across these and related studies (e.g., Alessi and Kahn 2019; Endres et al. 2015; Epp et al. 2022; Gilbert et al. 2012; Isobel, Gladstone, et al. 2021; Isobel, McCloughen, et al. 2021; Jacobowitz et al. 2015; Lietz et al. 2014; Mihelicova et al. 2018; Palfrey et al. 2019; Price et al. 2024; Rienecke et al. 2021; Rose et al. 2012; Shier and Turpin 2022; Thordarson and Rector 2020; Wilson et al. 2023; Wu et al. 2020; Xiao et al. 2016) shows that this hybridization is not merely eclectic, but rather a deliberate strategy to respond to the layered nature of trauma. Trauma has neurobiological dimensions (e.g., stress regulation, hyperarousal, dissociation), psychological and relational dimensions (e.g., attachment ruptures, mistrust, shame), and socio‐structural dimensions (e.g., discrimination, poverty, systemic violence). Consequently, single‐theory models risk being too narrow to inform meaningful service transformation. Another key reflection emerging from these studies is how hybrid frameworks support implementation fidelity and cultural adaptation. For example, Endres et al. (2015) used SAMHSA's organizational domains to structure system‐level trauma‐informed care transformation in Hawaiʻi, while simultaneously engaging local cultural values to sustain change (Endres et al. 2015). Palfrey et al. (2019) similarly highlight how brief TIC training rooted in broad theoretical principles can shift clinical practice, but sustained change requires embedding these principles within organizational structures (Palfrey et al. 2019). At the clinical level, Finch et al. (2024) link ACEs, attachment, and resilience to psychological distress among forensic populations, illustrating how explanatory models can guide targeted intervention (Finch et al. 2024). At the organizational and environmental level, Wilson et al. (2023) conceptualize trauma‐informed care through “relational, physical, and procedural security,” demonstrating that hybrid models can shape both practice and the built environment (Wilson et al. 2023). Critically, this pluralistic approach also introduces methodological and conceptual challenges. Without careful articulation, combining frameworks may blur theoretical boundaries and make evaluation more difficult. For example, studies often cite trauma‐informed principles without explicitly linking them to the underlying theoretical rationale (Rose et al. 2012; Wu et al. 2020), which can lead to variability in practice fidelity and hinder cross‐site comparison. This echoes broader concerns raised in the literature about the “conceptual looseness” of TIC (Sweeney et al. 2018; Thomas et al. 2019), which can either foster flexibility and local adaptation or lead to superficial implementation. However, the reflexive strength of hybrid approaches lies in their capacity to align with the lived complexities of mental health care. By integrating explanatory and implementation models, services can address trauma as both an individual and systemic phenomenon bridging neurobiological, psychological, social, and organizational dimensions. As trauma‐informed care moves from principle to practice, the field may benefit from more explicit articulation of how these frameworks are integrated, why certain models are chosen, and how they shape both intervention and evaluation strategies.

## 4 Discussion

### 4.1 Summary of Evidence

This scoping review provides an overview of the theoretical foundations that shape TIC in mental health settings. By mapping 23 included peer‐reviewed studies across seven thematic domains, the review highlights both the diversity and the convergence of theoretical approaches informing TIC. The findings reveal how neurobiological, psychological, socio‐ecological, and organizational frameworks intersect to address trauma as a multidimensional phenomenon. This synthesis offers important insights into how theory informs practice, implementation, and policy, and underscores the need for clearer theoretical articulation to strengthen the coherence and impact of TIC in mental health care.

### 4.2 Theoretical Advancements: Expanding the Conceptual Basis of TIC

One key contribution of this review is the clarification of TIC's theoretical heterogeneity. Early conceptualizations often framed TIC primarily through principles of safety, trust, and empowerment (Muskett 2014), but this review demonstrates that current scholarship increasingly integrates neurobiological, psychological, and socio‐structural frameworks. Neurobiological models, particularly polyvagal theory, advance understanding of trauma as an embodied phenomenon shaping stress response systems and relational safety (Wilson et al. 2023). Psychological and relational frameworks, especially attachment and psychodynamic theory, foreground trust, attunement, and relational repair as central mechanisms of recovery (Harris et al. 2025; Van Kranenburg et al. 2022). These models bring explanatory depth to TIC by specifying how and why trauma influences engagement, emotion regulation, and recovery trajectories. Simultaneously, ACE and social determinant frameworks (Finch et al. 2024; Wu et al. 2020) reframe trauma as a population‐level and structural determinant, shifting TIC from an individualized clinical intervention toward a universal, preventive approach. Intersectional and socio‐ecological perspectives further expand TIC's theoretical reach by situating trauma within broader systems of oppression, marginalization, and intergenerational harm (Thordarson and Rector 2020). This pluralism, while sometimes leading to conceptual ambiguity (Thomas et al. 2019), also represents a strength: it allows TIC to address the complex, multi‐level realities of trauma in contemporary mental health care.

### 4.3 New Insights Into Implementation and Practice

The review also highlights how theoretical models inform practice innovation and system change. SAMHSA's six principles remain the most widely operationalized framework, particularly for structuring organizational transformation (Endres et al. 2015; Palfrey et al. 2019). These principles provide a shared language across disciplines and facilitate alignment between clinical, organizational, and policy levels. Importantly, organizational and implementation science frameworks emphasize TIC as a systemic change process, requiring leadership engagement, workforce training, policy alignment, and environmental redesign (Rose et al. 2012; Shier and Turpin 2022; Wilson et al. 2023). This reflects a shift away from viewing TIC as an “add‐on” to care, toward conceptualizing it as an overarching organizational philosophy. Several studies illustrate how theoretical grounding supports concrete clinical strategies. For example, trauma‐informed training improved clinicians' confidence, increased trauma screening, and enhanced engagement (Palfrey et al. 2019). Trauma‐informed de‐escalation techniques reduced coercive practices and improved safety in inpatient settings (Price et al. 2024). TIC approaches embedded in addiction services improved retention and reduced retraumatization (Scoglio et al. 2020). These findings provide practical evidence that theory‐informed TIC implementation can yield measurable changes in service delivery and outcomes.

### 4.4 Bridging Individual and Structural Dimensions of Trauma

A significant insight from the review is the movement from individual pathology‐ and symptom‐focused models toward integrated frameworks that bridge neurobiological, relational, and structural understandings of trauma. Hybrid approaches are increasingly used to tailor TIC to diverse contexts, such as forensic services, addiction treatment, and crisis care (Harris et al. 2025; Finch et al. 2024; Goodkind et al. 2020). This integration responds to the layered nature of trauma: it is simultaneously embodied, interpersonal, and socially embedded. These hybrid models offer flexibility and cultural adaptability, though they also call for greater theoretical precision to ensure implementation fidelity and evaluability (Sweeney et al. 2018).

### 4.5 Cultural, Historical, and Violence‐Informed Dimensions

The review also exposes a cultural and conceptual gap in the TIC literature. Historical and intergenerational trauma, colonization, racism, displacement, and other forms of structural violence cannot be adequately understood if trauma is located only within the individual. A recent concept analysis published after the search underpinning this review extends this argument through the concept of trauma‐ and violence‐informed care, emphasizing the need to understand violence, power, and structural context alongside the effects of trauma (Isobel 2025). This perspective is particularly relevant to mental health nursing because apparently neutral routines, risk practices, language, and institutional rules can either reproduce powerlessness or create conditions for safety, choice, cultural humility, and shared decision‐making. Future framework development should therefore attend explicitly to Indigenous perspectives, refugee and asylum‐seeking experiences, racism‐related trauma, and culturally situated meanings of safety and recovery.

### 4.6 Implications for Mental Health Nursing

For mental health nurses, the findings move TIC beyond a generic statement of values. Neurobiological frameworks can support recognition of threat and arousal without automatically construing distress as pathology; relational models foreground attunement, trust, choice, and repair; socio‐ecological and intersectional approaches draw attention to power, racism, culture, gender, and structural violence; and implementation frameworks locate responsibility not only with individual nurses but also with nursing leadership, teams, policies, and care environments. In everyday practice, these perspectives support collaborative trauma inquiry, permission‐based communication, least‐coercive de‐escalation, reflective use of language, attention to environmental triggers, and advocacy when routines undermine dignity or choice. At the service level, mental health nurses can contribute to workforce learning, review of restraint and seclusion practices, trauma‐sensitive documentation, co‐design with service users, and evaluation of whether organizational policies actually enact trauma‐informed principles (Isobel and Edwards 2017; Isobel 2025).

### 4.7 Implications for Policy and System‐Level Transformation

The findings point to critical policy and system‐level implications. First, the high prevalence of trauma in mental health populations (Wu et al. 2020; Xiao et al. 2016) underscores the need for universal trauma‐informed practices, not just targeted interventions. Second, organizational structures must embed trauma‐awareness into core policies, documentation practices, and staff support systems to sustain change (Jacobowitz et al. 2015). Third, socio‐ecological and intersectional frameworks highlight that effective TIC must extend beyond the clinical setting to address structural inequities, including criminalization, racism, and gender‐based violence (Thordarson and Rector 2020). This requires cross‐sector collaboration and leadership commitment.

### 4.8 Contributions to Future Research

By mapping the theoretical terrain of TIC, this review lays groundwork for future research to examine the comparative value of different frameworks and develop integrated evaluation models. For example, linking neurobiological markers of safety with relational and organizational interventions may offer new ways to measure impact. Moreover, explicit articulation of how frameworks are combined in hybrid models can enhance reproducibility and transferability across settings.

### 4.9 Limitations

This review was designed and reported in accordance with PRISMA‐ScR recommendations. Multiple databases were searched, and the search strategy was developed iteratively in collaboration with an information specialist to capture the diverse theoretical and conceptual dimensions of trauma‐informed care. Data screening, extraction, and synthesis were conducted independently by two reviewers, with a third available for mediation. The main limitation of this review is the variability and, in some cases, limited explicitness of theoretical descriptions within the included studies. Many studies referred implicitly to trauma concepts without naming a specific framework, which required interpretive judgment during synthesis. Restricting the review to peer‐reviewed English‐language studies may also have excluded relevant contributions from non‐English or grey literature. As quality appraisal is not typically part of scoping review methodology, the review does not evaluate the rigor of included studies, and the prominence of certain frameworks reflects reporting frequency rather than theoretical strength.

## 5 Conclusion

This scoping review mapped the theoretical foundations of TIC in mental health settings, drawing on 23 peer‐reviewed studies. Seven overarching thematic domains were identified, reflecting the multidimensional nature of trauma as both an individual and structural phenomenon. The findings show that TIC is grounded in diverse frameworks—ranging from neurobiological and relational models to socio‐ecological and implementation science perspectives—underscoring its conceptual richness and complexity. Theoretical clarity is crucial for guiding implementation, enhancing organizational change, and strengthening mental health nursing and interdisciplinary practice. Embedding TIC principles into policies and everyday care can reduce traumatization, improve therapeutic relationships, and support recovery. Future research should focus on articulating hybrid frameworks and evaluating their impact at individual, organizational, and system levels to build more coherent and sustainable trauma‐informed mental health systems.

## 6 Relevance for Clinical Practice

This review provides mental health nurses with a framework for translating TIC from broad principles into clinical and organizational action. Neurobiological perspectives can help nurses understand arousal and threat responses without defaulting to pathologizing interpretations; relational frameworks foreground trust, choice, collaboration, and repair; and socio‐ecological and intersectional perspectives prompt attention to culture, racism, gender, historical trauma, and structural violence. In practice, this supports collaborative trauma inquiry, permission‐based communication, least‐coercive de‐escalation, reflective language, attention to environmental triggers, and shared decision‐making. At the organizational level, mental health nurses and nurse leaders can advocate for trauma‐sensitive documentation, review coercive practices, involve service users in co‐design, support staff reflection, and assess whether policies enact—rather than merely state—trauma‐informed principles. These actions connect nursing practice directly with recovery, dignity, safety, and cultural responsiveness.

## Author Contributions

Kim Jørgensen conceived the study and developed the review design. Kim Jørgensen and Maria Estrup Scredi conducted the screening, data extraction, and synthesis. Michael Haurum Marcussen acted as the third reviewer in cases of disagreement and contributed to the interpretation of the findings. All authors contributed to the critical revision of the manuscript, approved the final version, and accept responsibility for the integrity of the work.

## Funding

The authors have nothing to report.

## Ethics Statement

This study is a scoping review of published literature and did not involve human participants.

## Conflicts of Interest

The authors declare no conflicts of interest.

## Data Availability

The data that support the findings of this study are available from the corresponding author upon reasonable request.
